# The Nearly Complete Genome of *Grifola frondosa* and Light-Induced Genes Screened Based on Transcriptomics Promote the Production of Triterpenoid Compounds

**DOI:** 10.3390/jof11040322

**Published:** 2025-04-18

**Authors:** Yang Yang, Xuebo Hu

**Affiliations:** 1 Institute for Medicinal Plants, College of Plant Science and Technology, Huazhong Agricultural University, Wuhan 430070, China; life333@webmail.hzau.edu.cn; 2 Innovation Academy of International Traditional Chinese Medicinal Materials, Huazhong Agricultural University, Wuhan 430070, China

**Keywords:** *Grifola frondosa*, genome assembly, comparative genomics, transcriptome, triterpenoid metabolism

## Abstract

*Grifola frondosa*, commonly known as turkey tail, is a valuable fungus with medicinal and culinary uses, rich in bioactive compounds like triterpenoid polysaccharides that contribute to health benefits. Here, we constructed a nearly complete genome of *G. frondosa* CH1 using Illumina, PacBio HiFi, and Hi-C sequencing technologies, resulting in a 35.74 Mb genome with 12,526 protein-coding genes. The genome spans 12 chromosomes, all with intact telomeric structures and no gaps. The BUSCO completeness scores of 95.1% and 99.1% for the genome and genes, respectively, indicate high assembly quality and high completeness of gene prediction. Phylogenetic analysis showed a close relationship between *G. frondosa* CH1 and *Trametes cinnabarina*. Transcriptomic analysis under varying light conditions showed changes in the expression of genes, especially those related to terpenoid synthesis, with several CAZymes and CYP450 genes also exhibiting light-induced variations. Ten triterpenoid secondary metabolite gene clusters were identified, three of which were light-sensitive, indicating that light exposure regulates triterpenoid metabolism. This study provides valuable data supporting the high-quality genome of *G. frondosa* and offers new insights into the light-induced regulation of its metabolism.

## 1. Introduction

*Grifola frondosa,* also known as “Maitake” or “Hen-of-the-woods”, is a versatile mushroom used both medicinally and as food [1]. It is a fleshy, porous fungus classified under the Polyporaceae family within the phylum Stramenophora [2]. The mushroom is characterized by its large clusters of smoky brown rosette-shaped caps and its mature ascospores, which are dark grayish-brown in color and gradually fade as they age [3,4,5]. In nature, large clusters of ascospores are typically found at the base of tree stumps or on the ground near decaying hardwood trunks [6]. *G. frondosa* has been documented in various regions, including Japan, Europe, and high-altitude subtropical climates [7,8]. The species was first cultivated extensively in Japan, later spreading to China and the United States [9].

*G. frondosa* contains a variety of bioactive compounds, including polysaccharides, organic acids, alkaloids, coumarins, terpene lactones, and triterpenoids [10]. Pharmacological research has primarily focused on its bioactive polysaccharides [11]. The mycelium and fruiting bodies of *G. frondosa* are rich in carbohydrates, comprising 47% and 33% of the total weight, respectively, with water-soluble polysaccharides accounting for 3.8% [12]. To date, more than forty-seven polysaccharides have been isolated from *G. frondosa*, along with several biologically active fractions, including MD-fractions [13], MZ-fractions [14], SX-fractions [15], and Grifolans [16]. Notably, the MD-fraction, a protein-bound polysaccharide further purified from the D-fraction, has been developed over the past two decades as a complementary and alternative medicine for cancer treatment, as well as an alternative to the D-fraction [17]. It has been applied in complementary and alternative medicine and healthcare products for various cancers [18]. The SX-fraction, a glycoprotein, is commercially used to help regulate blood glucose levels and improve insulin sensitivity [19]. Furthermore, the China Food and Drug Administration (CFDA) has approved several patented drugs and health products containing SX-fraction glycoprotein as a key ingredient [20]. Pharmacological studies have shown that crude polysaccharides from *G. frondosa* exhibit various biological activities, including antitumor, immunomodulatory, antioxidant, hepatoprotective, and antihyperglycemic effects [21].

Besides polysaccharides, triterpenoids in *G. frondosa* have been identified as valuable bioactive compounds with significant roles in the treatment and prevention of cancer, neurodegenerative diseases, and human immunodeficiency virus (HIV) [22]. Moreover, triterpenoids are biosynthesized in fungi, leading to the production of sterols that serve as the foundational framework for eukaryotic cells [23].

Here, we obtained a high-quality chromosome-level genome of *G. frondosa* CH1 through Illumina and PacBio sequencing. Comparative genomic analysis revealed the evolutionary relationships, commonalities, and differences between *G. frondosa* CH1 and other closely related species. Furthermore, we investigated the metabolic and expression differences of polysaccharides and triterpenoids in *G. frondosa* CH1 and explored the specific effects of light on the production of these bioactive compounds. This study provides crucial data support for research on the *G. frondosa* CH1 genome and key bioactive substances.

## 2. Materials and Methods

### 2.1. Culture Conditions for G. frondosa CH1

The *G. frondosa* CH1 strain was isolated from Liaoyang City, Liaoning Province, China, and cultured on Potato Dextrose Agar (PDA) medium. The PDA medium was prepared by dissolving 220 g of potato, 18 g of glucose, and 18 g of agar per liter of distilled water. The cultures were incubated at 28 °C until the mycelium had fully colonized the surface of the Petri dish. Once the entire surface was covered, the mycelium was transferred to fresh PDA plates and incubated for 10 days prior to further experiments.

### 2.2. DNA Extraction and Genome Sequencing

Following a standardized protocol, genomic DNA from *G. frondosa* CH1 was extracted using the Sangon Biotech Quick Fungal DNA Kit (Catalog No. B518229-0050). The quality and purity of the extracted DNA were assessed using 0.5% agarose gel electrophoresis and the NanoDrop 2000 spectrophotometer (Thermo Scientific, Wilmington, DE, USA). High-quality whole-genome sequencing was obtained by combining Illumina and PacBio sequencing technologies. A short-read library (insert size 300 bp) was constructed on the Illumina HiSeq X platform (Illumina Inc., San Diego, CA, USA), and a long-read library (insert size 20 kb) was constructed on the PacBio Revio platform (Pacific Biosciences, Menlo Park, CA, USA). Chromosome-level genome assembly was performed using Hi-C sequencing in paired-end mode (PE150) on the Illumina HiSeq X platform, providing high-quality Hi-C data. All sequencing work was completed by Frasergen Bioinformatics (Wuhan, China).

### 2.3. Genome Assembly

The HiFi sequences obtained from PacBio CCS sequencing of the *G. frondosa* genome were assembled using Hifiasm (v0.19.9) [24] software with default parameters. The initial assembly was then corrected with Illumina short reads using NextPolish (v1.4.1) [25], resulting in a complete contig-level genome assembly for *G. frondosa* CH1. Clean reads from Hi-C sequencing were aligned to the *G. frondosa* CH1 genome using ALLHiC (https://github.com/tangerzhang/ALLHiC), and the contigs were subsequently anchored to chromosomes with 3D-DNA (v180922) [26]. Manual corrections of the anchoring results were conducted using Juicebox (v1.13) [27], culminating in the final chromosome-level genome assembly of *G. frondosa* CH1, with all software running on default parameters. The integrity of the assembled genome was assessed using BUSCO (v5.1.2) [28], comparing it to the fungal lineage dataset fungi_odb10.

### 2.4. Gene Prediction and Annotation

Repetitive sequences in the genome were identified using RepeatModeler (v2.0.5) [29] and RepeatMasker(v1.0) [30]. First, de novo structural prediction was performed with RepeatModeler to construct a species-specific library of repetitive sequences. This library was then imported into RepeatMasker for further analysis to identify and mask regions like known repetitive sequences in the genome. Additionally, the RepBase database was utilized to predict genomic fragments resembling known repeats, enhancing the accuracy and comprehensiveness of the analysis.

Gene model prediction for the *G. frondosa* CH1 genome was performed using the MAKER pipeline [31]. Initially, de novo gene prediction was carried out with Augustus (v2.4) [32] and SNAP (v1.0) [33] to generate an initial set of gene models. Homologous gene prediction was further refined using GeMoMa (v1.9) [34], which enhanced and validated the accuracy of the gene models based on homologous information. Finally, various predicted gene sets were integrated into a non-redundant and more complete gene set using EVidenceModeler (v1.1) [35].

Functional annotation of the predicted gene models was achieved by comparing them to gene and protein sequences in the NCBI nucleotide database (Nt) and UniProt/Swiss-Prot database. Protein domains of the gene models were annotated using InterProScan (v5.59.91.0), and all genes were classified based on Gene Ontology (GO), the Eukaryotic Orthologous Groups (KOG) database, and the KEGG metabolic pathway database to explore their biological functions and metabolic pathways. The prediction of snRNA was performed using Infernal (v1.1.2) [36], and alignment with the Rfam database was employed to detect rRNAs and microRNAs. tRNA models were predicted using tRNAscan (v2.0.9) [37], while the identification of rRNA was completed using BLASTn (v2.7.1).

### 2.5. Gene Family Clustering and Phylogenetic Analysis

To investigate the evolutionary characteristics of gene families in the *G. frondosa* CH1 genome, genomic sequences of a total of 12 fungi (*Phanerochaete hrysosporium*, *Gelatoporia ubvermispora*, *Schizophyllum commune*, *Fomitopsis pinicola*, *Daedalea quercina*, *Trametes cinnabarina*, *Polyporus arcularius*, *Dichomitus squalens*, *Lentinula edodes*, *Ganoderma lucidum*, *Wolfiporia cocos*) were obtained from the NCBI database. These species are representative members of the Polyporales, with their genomic data publicly available. They exhibit phylogenetic diversity and share a close relationship with *G. frondosa*, facilitating a more reliable inference of gene family evolutionary trends. OrthoFinder (v2.5.4) [38] was utilized to perform clustering analysis for orthologous genes and gene families based on the protein sequences of the species. Single-copy genes obtained from clustering were subjected to multiple sequence alignment using MAFFT (v7.471) [39]. The ModelFinder module was utilized to automatically select the optimal substitution model, and the maximum likelihood method was applied to construct the phylogenetic tree using IQ-TREE (v2.0.6) [40] with 1000 bootstrap replicates. Divergence times were obtained from the TimeTree website (http://www.timetree.org/), and the mcmctree package in PAML (v4.10.7) [41] was used to estimate species divergence times. Based on the species phylogenetic tree, gene family expansions and contractions were identified using the CAFÉ (v4.2.1) [42]. GO and KEGG enrichment analyses were conducted on the expanded and contracted gene families using the R package clusterProfiler (v4.14.0).

### 2.6. Comparative Genomic Analysis

Interspecies genomic relatedness was assessed through pairwise average nucleotide identity (ANI) computations performed with fastANI (v1.34), employing a k-mer-based alignment-free algorithm [43]. Gene synteny analysis was conducted using MCScanX [44]. BLASTp (E < 1 × 10^−5^) was employed for both all-vs-all and self-comparisons to identify syntenic regions between different species. The synteny results were visualized using JCVI (v1.4.16).

### 2.7. Transcriptome Analysis

Mycelia of *G. frondosa* CH1 were collected during the growth phase and exposed to two conditions for 7 days: complete darkness (control group) and normal light (treatment group). Three biological replicates were prepared for each condition. These replicates were derived from independent fermentations cultured under identical conditions to ensure reproducibility. Within each biological replicate, Petri dishes were inoculated from the same starting culture. Prior to sequencing, we evaluated mycelial growth under both dark and light conditions. Specifically, mycelia were cultured at 28 °C under complete darkness or continuous light, with colony diameters measured every 24 h in both conditions. Statistical analysis confirmed that mycelial growth was comparable between the two conditions (Figure S1). Following treatment, mycelia were promptly harvested, flash-frozen in liquid nitrogen, and stored at −80 °C for RNA extraction. RNA was extracted following a previously established protocol [45], and cDNA libraries were subsequently constructed. These libraries were sequenced on the Illumina HiSeq X platform. Post sequencing, raw data were processed using FastQC (v0.12.1) for quality control, and adapters along with low-quality reads were removed using fastp (v0.23.4) [46]. Cleaned reads were aligned to the *G. frondosa* CH1 genome using HISAT2 (v2.2.1) [47] with default parameters. Aligned reads were assembled with StringTie (v2.2.1) [48], and gene expression levels were quantified as FPKM (Fragments Per Kilobase of transcript per Million mapped reads) values using Cufflinks (v2.2.1) [49]. Differentially expressed genes (DEGs) were identified based on a fold change (FC) ≥ 2 or ≤0.5, with a statistical significance threshold of *p* < 0.05.

### 2.8. Identification of CAZymes, CYP450 and Secondary Metabolic Clusters

First, the P450 domain model (Pfam ID: PF00067) was downloaded from the Pfam database (http://pfam.xfam.org/ (accessed on 20 November 2024)). Subsequently, HMMER 3.0 was used to search all *G. frondosa* CH1 protein sequences based on this P450 reference domain model with a threshold of (E < 1 × 10^−5^). The initially screened CYP450 genes were then submitted to the SMART database (https://smart.embl.de/ (accessed on 20 November 2024)) for manual identification. Finally, the identified P450 genes were confirmed as the cytochrome P450 genes of *G. frondosa* CH1.

Except for the *G. frondosa* genome sequence generated in this study, the genome sequences of three additional species used for comparative genomics were retrieved from NCBI, with GenBank accession numbers GCA001683735.1, GCA003313135.1, and GCA041464085.1. To standardize the annotation of CAZymes across the 4 *G. frondosa* genomes, we downloaded protein sequences and characteristic domains of key CAZymes, including auxiliary activities (AAs), carbohydrate esterases (CEs), polysaccharide lyases (PLs), glycoside hydrolases (GHs), and glycosyltransferases (GTs) from the CAZy database (http://www.cazy.org (accessed on 12 October 2024)). CAZymes in *G. frondosa* CH1, GCA001683735.1, GCA003313135.1, and GCA041464085.1 were subsequently predicted using a combination of homology-based alignment and domain searches. For homology-based prediction, BLASTP was employed with the downloaded CAZymes protein sequences as references, identifying candidate CAZymes proteins in the 4 genomes with an e-value threshold of <1 × 10⁻^5^. Next, HMMER was used to search for candidate CAZymes based on the characteristic domains of key CAZymes, selecting genes with an e-value ≤1 × 10⁻^5^ as CAZymes. Proteins matching the specific reference CAZymes domains were then classified as corresponding CAZymes genes.

### 2.9. qRT-PCR Analysis

To validate the reliability of transcriptome sequencing data, 8 genes (*FUN_001792*, *FUN_002153*, *FUN_001933*, *FUN_001956*, *FUN_001494*, *FUN_011564*, *FUN_005912*, and *FUN_006226*) were randomly selected for verification. Total RNA purification from fungal cultures was achieved through a fungal-specific RNA extraction protocol (Fungal Total RNA Isolation Kit, manufacturer-specified workflow). RNA from different treatment groups was reverse-transcribed into first-strand cDNA, and gene-specific primers were designed using Primer Premier 5.0 (Table S1). Gene expression levels were quantified by quantitative PCR (qPCR) using the 2^−ΔCt^ method, where ΔCt was calculated as the difference between the cycle threshold (Ct) value of the reference gene and that of the target gene. We selected *actin* (*FUN_000155*) as the normalized reference gene and validated its expression stability under our experimental conditions using qRT-PCR (Figure S2). Fold changes in expression between experimental and control groups were analyzed using the 2^−ΔΔCt^ method, where ΔΔCt represents the difference in ΔCt values between the experimental and control groups. Statistical analysis was performed using a *t*-test, with significance set at *p* < 0.05, and error bars represent the standard deviation (SD).

## 3. Results

### 3.1. Genome Assembly Analysis

**Figure 1:**
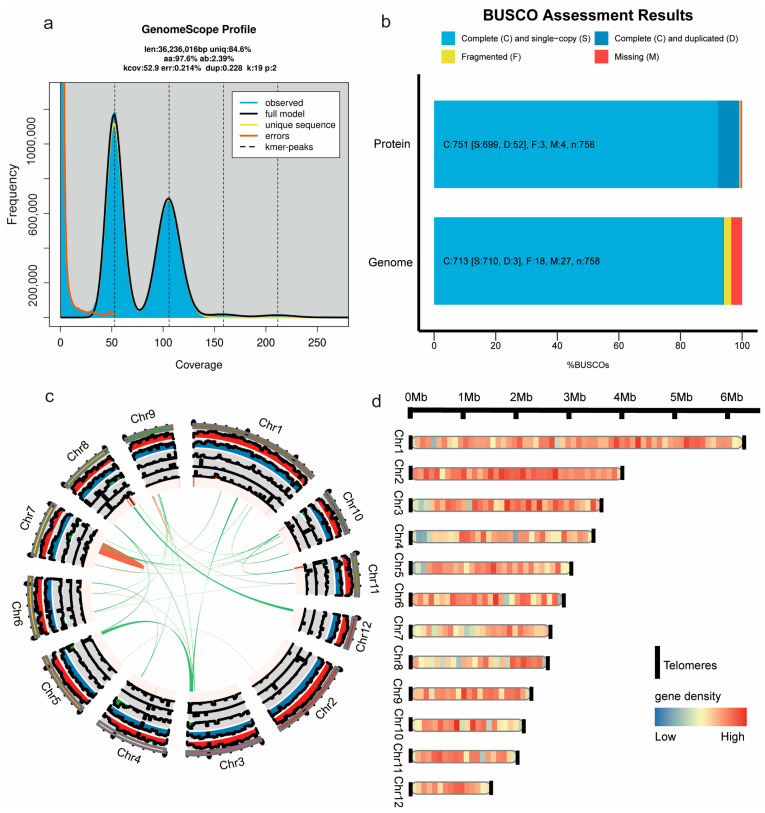
Genome assembly of *G. frondosa* CH1. (**a**) The k-mer distribution results estimating the genome size of *G. frondosa* CH1. (**b**) Assesses the completeness of the *G. frondosa* CH1 genome using the BUSCO method with fungal and basidiomycete datasets. The color codes represent single-copy genes (light blue), multi-copy genes (dark blue), fragmented genes (yellow), and missing genes (red). (**c**) Chromosome circle diagram of *G. frondosa* CH1, with the outermost length representing chromosome segment size, and the interior representing GC content and gene density, respectively. Green lines indicate segmental duplications, and red lines indicate tandem duplications. (**d**) Chromosome arrangement diagram of *G. frondosa* CH1, chromosome length indicates the size of the gene, flanked by telomeres, and the arrangement of colors on the strip indicates the density of the gene, with red being the higher density and blue being the lower density.

To obtain a high-quality genome assembly of *G. frondosa* CH1, sequencing was performed using both PacBio Revio and Illumina HiSeq X high-throughput platforms. Illumina sequencing generated approximately 5.68 Gb of data, with a coverage depth of about 160× and an average Q30 value of 93.25% (Table S2). PacBio HiFi sequencing produced 8.51 Gb of data, with an average read length of 18.50 kb and a sequencing depth exceeding 230× (Table S3). Hi-C sequencing provided 18.74 Gb of data, with an average Q30 value of 95.66% and a sequencing depth of approximately 520×. All three sequencing platforms produced high-quality data, significantly enhancing the accuracy of the *G. frondosa* CH1 genome assembly (Table S4). Using Illumina sequencing data and K-mer analysis, the genome size of *G. frondosa* was initially estimated to be around 36.2 Mb (Figure 1a). The genome was subsequently assembled using PacBio HiFi long-read data, with redundancy removal and error correction performed using Illumina short reads. The final assembly resulted in a genome size of 38.8 Mb, consisting of 30 contigs, with an N50 of 2.98 Mb and a GC content of 49.78% (Table S5). To achieve a chromosomal-level assembly, Hi-C data were incorporated, successfully anchoring the contigs onto 12 chromosomes. The Hi-C interaction heatmap revealed strong interaction signals between loci within each chromosome, confirming the accuracy of the chromosomal-level assembly (Figure S3). The final chromosomal-level assembly of the *G. frondosa* CH1 genome has a size of 35.74 Mb, an N50 of 2.98 Mb, and a contig mapping rate of 92.08% (Table S6).

The BUSCO evaluation of the assembled *G. frondosa* CH1 genome revealed a completeness score of 94.1%, with 93.7% of the BUSCO genes present as complete single-copy genes, 0.4% as complete duplicated genes, 2.4% as fragmented genes, and 3.6% as missing genes, further confirming the high quality of the genome assembly (Figure 1b). The genome circular plot reflects the overall characteristics of the assembled *G. frondosa* CH1 genome, including the density of Copia and Gypsy retrotransposons, simple repeat sequences, gene density, GC content, intra-species homology regions, and chromosome length information (Figure 1c). To achieve a more complete genome assembly, telomeric regions (AACCT) on all 12 chromosomes were successfully identified. The distribution of gene density across chromosomes indicated that chromosome 2 has a higher gene density, with genes primarily concentrated in the central region of the chromosome (Figure 1d). The final genome assembly, from telomere to telomere, demonstrates the accuracy and completeness of the *G. frondosa* CH1 genome.

### 3.2. Genome Annotation

The genome of *G. frondosa* CH1 contains approximately 13.48% repetitive sequences, the majority of which are LTR retrotransposons, particularly Copia and Gypsy, which account for 1.36% and 2.61% of the genome length, respectively (Table S7). Gene prediction was conducted using a combination of de novo prediction, homology-based alignment, and transcriptome assembly, resulting in the identification of 12,526 protein-coding genes with an average length of 1904 base pairs. The genome of *G. frondosa* CH1 contains a total of 74,833 coding sequences (CDS) and 78,721 exons, with average lengths of 217.12 base pairs and 6.28 base pairs, respectively. On average, each gene contains 5.97 CDS and 6.28 exons. Additionally, 66,358 introns were identified, with an average length of 63.58 base pairs (Table 1). The completeness of the predicted gene dataset was 99.1%, with 92.2% of the BUSCO genes being complete single-copy genes, 6.9% as complete duplicates, 0.4% as fragmented, and 0.5% as missing. These results validate the high integrity of the predicted gene set. Non-coding RNA (ncRNA) prediction revealed that *G. frondosa* CH1 contains 342 tRNA genes, 26 small nuclear RNA (snRNA) genes, 7 small nucleolar RNA (snoRNA) genes, and 1 small RNA (sRNA) gene (Table S8).

**Figure 2:**
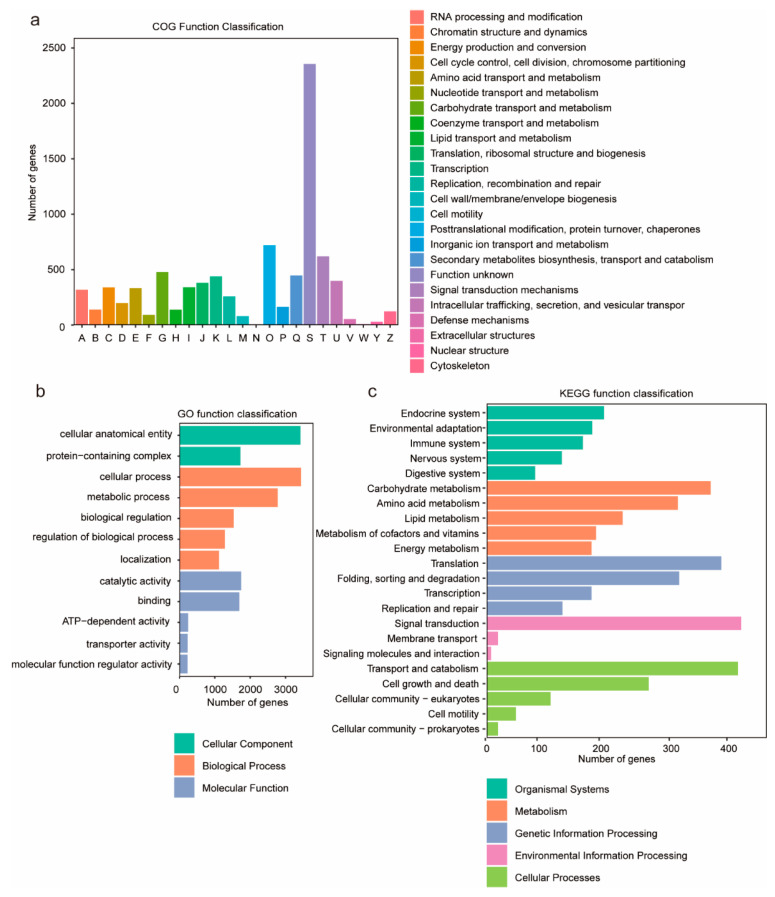
The graphical representations depict gene annotation analyses of *G. frondosa* CH1. (**a**) COG functional classification: The *x*-axis denotes functional categories of genes, while the *y*-axis indicates the quantitative distribution of genes within each functional category. (**b**) GO annotation enrichment: The top 12 significantly enriched entries are presented, with color-coded categorization corresponding to Biological Process (BP), Cellular Component (CC), and Molecular Function (MF). (**c**) KEGG pathway annotation: The *y*-axis enumerates metabolic pathway designations, whereas the *x*-axis quantifies the number of annotated genes per pathway. KEGG metabolic pathways are systematically classified into seven principal domains: metabolism, genetic information processing, environmental information processing, cellular processes, organismal systems, human diseases, and drug development.

To investigate the biological functions of the predicted genes, functional annotation was performed using the NR, COG, GO, and KEGG databases. The results indicated that a total of 11,849 genes were functionally annotated, representing 94.6% of all predicted genes. Functional categorization of the annotated genes was performed using the eggNOG database (Figure 2a), and the results showed that the category “signal transduction mechanisms” contained the largest number of genes, comprising 2368 genes. GO enrichment analysis showed that 3630 genes (29.36%) were annotated in the GO database (Table S9), and the most abundant GO terms were “cellular anatomical entity” and “cellular process”, followed by “metabolic process” and “catalytic activity” (Figure 2b). Furthermore, 2837 genes (22.95%) were annotated to KEGG pathways, categorized into five primary classifications: “environmental information processing”, “cellular processes”, “genetic information processing”, “organismal systems”, and “metabolism”. Among these, the categories with the highest proportions of annotated pathways were “environmental information processing” and “cellular processes”. Within these two categories, the secondary pathways “signal transduction” and “transport and catabolism” were the most frequently represented by genes (Figure 2c).

### 3.3. Genomic Comparison of Different G. frondosa Genome Assemblies

**Figure 3:**
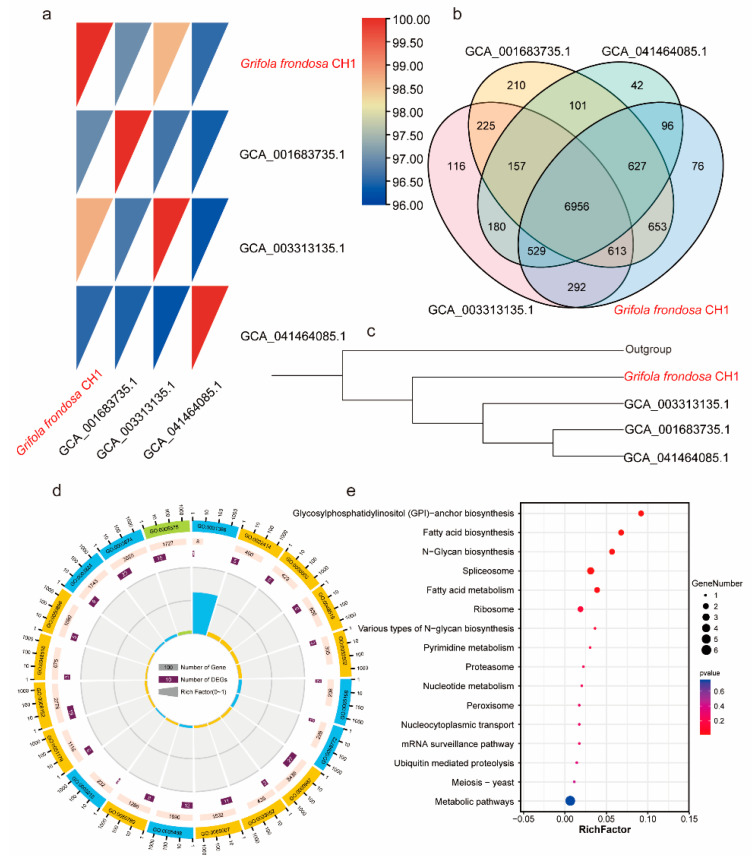
Comparative genomics analysis of the four *G. frondosa* genomes: (**a**) The ANI (average nucleotide identity) heatmap shows pairwise genomic comparisons of all *G. frondosa* CH1 strains. (**b**) The Venn diagram illustrates the shared and unique gene families among the four *G. frondosa* genomes. (**c**) A phylogenetic tree constructed using the four *G. frondosa* genomes with GCA_041464085.1 as the outgroup. (**d**) GO enrichment analysis of unique genes in the *G. frondosa* CH1 genome. (**e**) KEGG enrichment analysis of unique genes in the *G. frondosa* CH1 genome.

Prior to this study, three genomes of *G. frondosa* had been sequenced and published, with GenBank accession numbers GCA001683735.1, GCA003313135.1, and GCA041464085.1. To investigate the differences between the genome of *G. frondosa* CH1 sequenced in this study and the previously published genomes, we analyzed their average nucleotide identity (ANI). The results revealed that *G. frondosa* CH1 shared the highest ANI (98.5%) with GCA003313135.1, followed by GCA001683735.1 (97%) and GCA041464085.1 (96.5%). These findings indicate that *G. frondosa* CH1 has a closer phylogenetic relationship with GCA003313135.1 (Figure 3a). Cluster analysis showed that the four genomes share a total of 6956 gene families, with 76 unique gene families identified in *G. frondosa* CH1 (Figure 3b). Furthermore, phylogenetic tree analysis revealed that GCA001683735.1, GCA003313135.1, and GCA041464085.1 are more closely related to each other than to *G. frondosa* CH1, with GCA003313135.1 being most closely related to *G. frondosa* CH1, consistent with the ANI results (Figure 3c). GO enrichment analysis of 76 unique gene families (comprising 101 genes) in the *G. frondosa* CH1 genome showed significant enrichment in the “reproductive process” and “cellular process” categories (Figure 3d). KEGG pathway analysis suggested that these unique genes may be involved in the “glycosylphosphatidylinositol (GPI)-anchor biosynthesis” and “fatty acid biosynthesis” pathways (Figure 3e). Furthermore, based on the analysis of whole-genome collinearity, it was found that the *G. frondosa* CH1 exhibits a high degree of linkage conservation with another *G. frondosa* sampled in Baoding, China, specifically GCA_041464085.1. For instance, the linkage regions of *G. frondosa* CH1’s chr1 and chr12 show significant conservation. Additionally, there are instances of chromosomal fusion; for example, the linkage region on *G. frondosa* CH1’s chr1 corresponds to both chr1 and chr7 in GCA_041464085.1 (Figure S4).

### 3.4. Phylogenetic Analysis of G. frondosa CHI and Other Fungi

**Figure 4:**
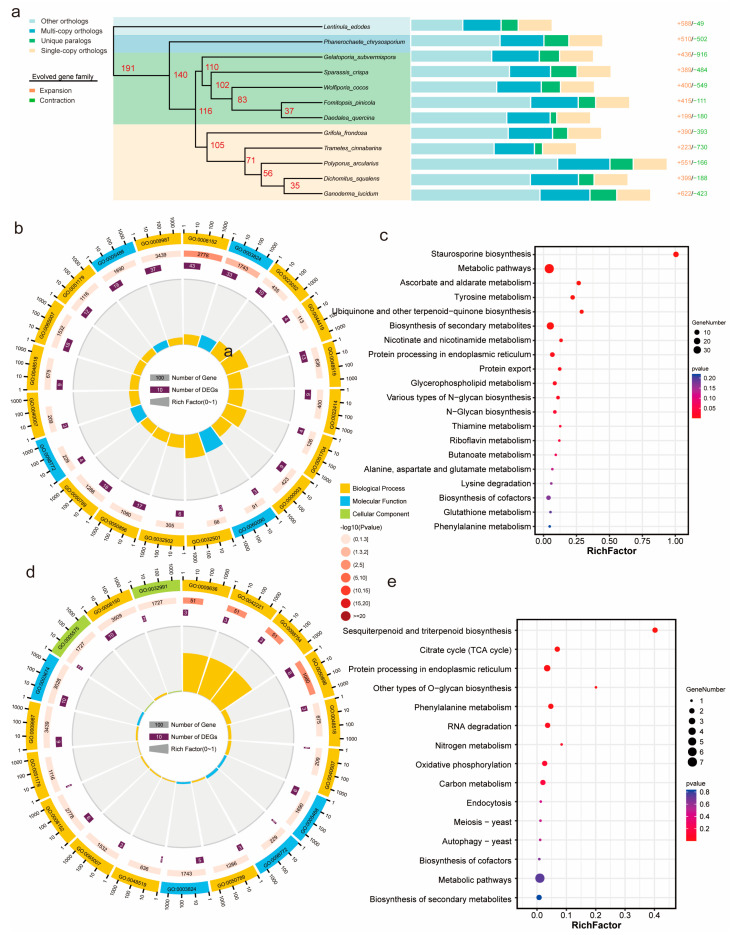
Phylogenetic analysis of *G. frondosa* CH1 and other fungi: (**a**) The phylogenetic relationship of *G. frondosa* CH1 with 11 representative basidiomycetes, using *L. edodes* as the outgroup species. The bar chart represents the gene family distribution types for each species, with numbers indicating the expanded and contracted gene families in each species, where red represents expansion and green represents contraction. (**b**) GO enrichment analysis of expanded genes in the genome of *G. frondosa* CH1. (**c**) KEGG enrichment analysis of expanded genes in the genome of *G. frondosa* CH1. (**d**) GO enrichment analysis of contracted genes in the genome of *G. frondosa* CH1. (**e**) KEGG enrichment analysis of contracted genes in the genome of *G. frondosa* CH1.

To investigate the evolutionary relationship of *G. frondosa* CH1 among polypore fungi, we selected 10 other polypore species and used *Lentinula edodes* as the outgroup for gene family clustering analysis. The results revealed a total of 158,596 genes across the 12 species, with 13,817 orthogroups identified, containing 140,045 genes (88.3%), and 18,551 genes (11.7%) were not grouped into any orthogroup. Among these orthogroups, 2009 gene families were classified as single-copy genes (Table S10). To better understand the phylogenetic relationship of *G. frondosa* CH1 with other fungi, we performed multiple sequence alignment of the 2009 single-copy genes and constructed a phylogenetic tree (Figure 4a). The phylogenetic analysis indicated that the 12 fungi can be broadly divided into three groups: the ancient polypores represented by *Phanerochaete chrysosporium*, the second group of polypores represented by *Wolfiporia cocos*, and the third group represented by *Ganoderma lucidum*. *G. frondosa* CH1 belongs to the third group of polypores, and phylogenetically, it is most closely related to *Trametes cinnabarina*.

To estimate the divergence time of *G. frondosa* CH1, we used fossil calibration points and estimated the divergence rates based on fungal evolutionary studies. The results showed that the divergence between the third and second groups of polypores occurred approximately 116 million years ago (Mya), while the divergence between *G. frondosa* CH1 and *T. cinnabarina* occurred around 105 Mya. Gene family contraction and expansion analysis based on the phylogenetic tree revealed that 390 gene families were significantly expanded, and 393 gene families were significantly contracted in *G. frondosa* CH1. GO enrichment analysis of these gene families revealed that expanded gene families were significantly enriched in functional categories related to “metabolic processes” and “catalytic activity” (Figure 4b), whereas contracted gene families were primarily enriched in categories associated with “cellular processes” and “binding” (Figure 4d). KEGG enrichment analysis indicated that expanded gene families were significantly enriched in metabolic pathways such as “staurosporine biosynthesis” and “biosynthesis of secondary metabolites” (Figure 4c), while contracted gene families were mainly enriched in pathways including “sesquiterpenoid and triterpenoid biosynthesis” and the “citric acid cycle (TCA cycle)” (Figure 4e).

### 3.5. Transcriptome of G. frondosa CHI Under Light and Dark Conditions

**Figure 5:**
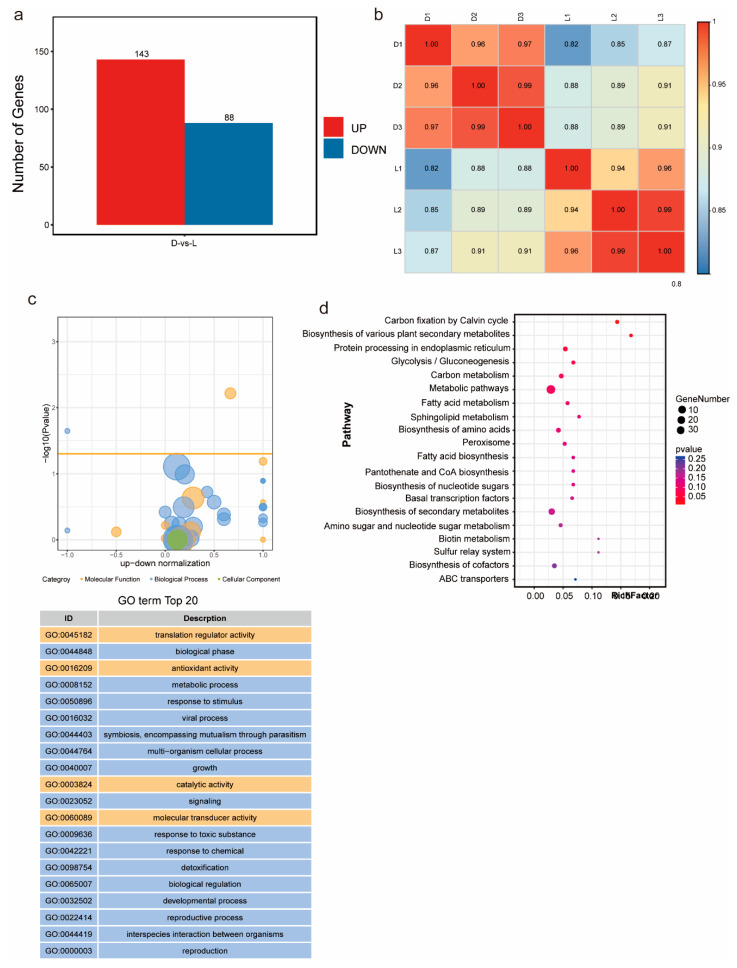
Transcriptomic analysis of *G. frondosa* CH1 was conducted under two different light cycle treatments: standard light intensity and complete darkness, with a 7-day experimental duration. (**a**) Compared to the complete darkness condition (control group), the number of differentially expressed genes (DEGs) under standard light intensity is shown, with red bars representing the number of upregulated genes and blue bars representing the number of downregulated genes. (**b**) Transcriptional correlation analysis between samples, represented by the Pearson correlation coefficient (R^2^). (**c**) The top 20 significantly enriched GO terms for the differentially expressed genes. (**d**) KEGG enrichment analysis of DEGs, where the size of the circles corresponds to the number of enriched genes, and the color gradient represents the statistical significance level.

To explore the molecular mechanisms of metabolic regulation in *G. frondosa* CH1 under both dark and light conditions, we conducted transcriptome sequencing on samples collected from *G. frondosa* grown in complete darkness (three biological replicates) and under standard light intensity (three biological replicates), generating a total of 17 Gb of clean data (Table S11). Differential expression analysis identified 231 significantly differentially expressed genes (DEGs), including 143 upregulated genes and 88 downregulated genes (Figure 5a). Pearson correlation analysis showed that the R^2^ values for all samples, except for L1, were greater than 0.85, indicating strong correlation among the samples (Figure 5b). GO enrichment analysis revealed that the differentially expressed genes were significantly enriched in terms related to “biological processes”, followed by “molecular functions”. Statistical analysis of the top 20 GO terms found that “translation regulator activity”, “antioxidant activity”, “catalytic activity”, and “molecular transducer activity” were the most prominent in molecular functions (Figure 5c). The remaining GO enrichment pathways were mainly associated with biological processes. KEGG enrichment analysis showed that “metabolic pathways” and “biosynthesis of secondary metabolites” were the most significantly enriched pathways (Figure 5d). These results indicate that light and dark conditions significantly impact the synthesis and metabolism of secondary metabolites in *G. frondosa* CH1.

### 3.6. Carbohydrate-Active Enzymes May Be Involved in Regulating Metabolic Synthesis

**Figure 6:**
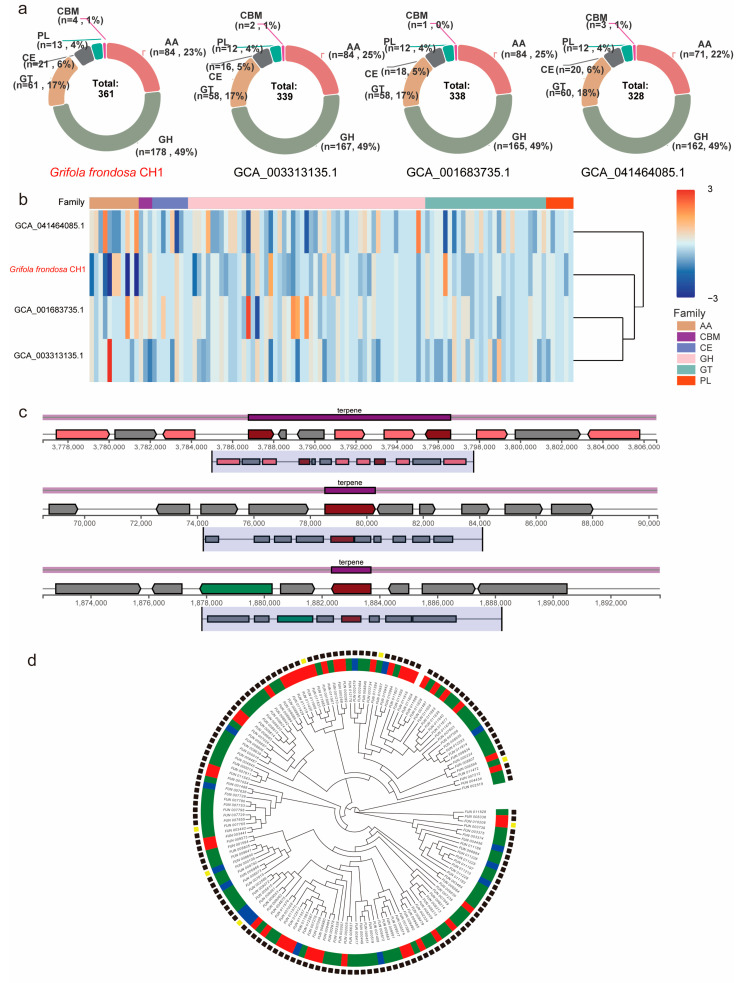
The distribution patterns and expression profiles of CAZymes (Carbohydrate-Active Enzymes) genes across four *G. frondosa* genomes. (**a**) Comparative analysis of CAZymes gene distribution among the following genomic assemblies: *G. frondosa* CH1, GCA001683735.1, GCA003313135.1, and GCA041464085.1. (**b**) Differential expression patterns of CAZymes genes in *G. frondosa* strains, namely, CH1, GCA001683735.1, GCA003313135.1, and GCA041464085.1. (**c**) The structural organization of DEGs within triterpenoid metabolic gene clusters in *G. frondosa* CH1 under controlled photoperiod conditions. (**d**) Phylogenetic tree of 361 CAZyme genes in *G. frondosa* CH1. The concentric circles represent the classification of genes into different subfamilies, with the outer and inner rings corresponding to different CAZyme subfamily classifications. In the outer group, lines in different colors represent distinct CYP450 gene families.

Lignin is one of the key structural components of plant cell walls and is the third most abundant renewable carbon source in nature, following cellulose and hemicellulose. Fungi degrade lignin into low molecular weight aromatic compounds by secreting lignin-degrading enzymes (such as laccase, lignin peroxidase, and manganese peroxidase). These compounds are further metabolized by the fungi, serving as a carbon source and energy supply. Under dark conditions, differential expression of CAZyme genes is closely associated with enhanced lignin degradation capacity and may facilitate sclerotium development. To investigate the CAZyme characteristics in *G. frondosa* CH1, we identified its carbohydrate-active enzymes (CAZymes). A total of 361 lignin-degrading enzymes were successfully identified in the *G. frondosa* CH1 genome, with glycoside hydrolases (GHs) comprising 49%, auxiliary activities (AAs) 23%, glycosyltransferases (GTs) 17%, carbohydrate esterases (CEs) 6%, polysaccharide lyases (PLs) 4%, and carbohydrate-binding modules (CBMs) 1% (Table S12). Compared to the other three assembly versions of *G. frondosa*, the *G. frondosa* CH1 genome contains a greater total number of CAZyme genes, although the proportions of enzyme families do not differ significantly (Figure 6a). Among the four species, GHs constitute the highest proportion (approximately 49%), followed by AAs (23%–25%) (Table S13). Moreover, the expression pattern of auxiliary activities in the *G. frondosa* CH1 genome differs from the other three genomes, with significantly lower expression levels (Figure 6b). These enzymes may play an important regulatory role in the biosynthesis of secondary metabolites in *G. frondosa* CH1.

CYP450 is one of the key enzymes involved in triterpene modification in fungi. To investigate the effect of light on metabolite synthesis in G. frondosa CH1, a total of 149 CYP450 genes were identified in the *G. frondosa* CH1 genome. Phylogenetic analysis classified these genes into six major groups: CYP5139, CYP5154, CYP63, CYP5035, CYP512, and CYP5037. Among them, the CYP5037 subfamily contained the largest number of genes, followed by CYP5139. Further analysis of transcriptomic data revealed that 43 CYP450 genes were significantly upregulated under light conditions, while only 15 genes showed higher expression levels in darkness than under light. These results suggest that light may promote the expression of most CYP450 genes (Figure 6c).

To investigate the direct relationship between secondary metabolism and light in *G. frondosa* CH1, we identified 24 secondary metabolism gene clusters in its genome, including 10 triterpene biosynthesis clusters, 10 non-ribosomal peptide synthetase (NRPS) clusters, 2 T1PKS clusters, and 2 other secondary metabolism clusters (Table S14). Among the 10 triterpene metabolism clusters, 3 were found to contain significant photoreceptor genes, suggesting that triterpene metabolism may be associated with light conditions. Light environment could be one of the factors influencing these triterpene metabolism clusters (Figure 6d).

### 3.7. qRT-PCR Validation of Differential Expression

**Figure 7:**
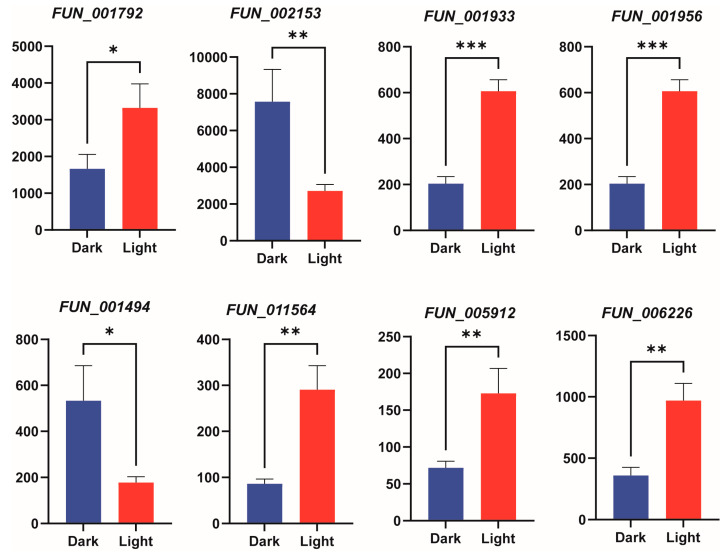
qRT-PCR validation of gene expression under light treatment. The bar chart shows the expression levels of 8 genes. Bars represent the mean ± standard deviation of three biological replicates. Bar charts depict gene expression levels under different treatment conditions, with statistical significance between treatments denoted by asterisks: * indicates *p* < 0.05, ** indicates *p* < 0.01, and *** indicates *p* < 0.001.

To validate the reliability of the transcriptomic data, we randomly selected 8 genes related to the terpenoid metabolic pathway (*FUN001792*, *FUN002153*, *FUN001933*, *FUN001956*, *FUN001494*, *FUN011564*, *FUN005912*, *FUN006226*) for qRT-PCR verification. The results showed that the expression patterns obtained from qRT-PCR were consistent with the RNA-seq data, confirming the reliability of the transcriptomic analysis (Figure 7).

## 4. Discussion

*G. frondosa*, belonging to the Basidiomycota phylum (class Agaricomycetes, order Polyporales), is an important species with both culinary and medicinal value. Its fruiting bodies are increasingly sought after for their palatability and diverse pharmacological properties, including antitumor [50], immunomodulatory [51], hypoglycemic [52], and antiviral activities [53], as well as their potential to promote beneficial gut microbiota. Research has highlighted the pivotal roles of polysaccharides and triterpenoids in its medicinal effects. Notably, external factors such as light exposure significantly influence mycelial growth and regulate the biosynthesis and metabolism of these bioactive compounds.

Currently, the NCBI GenBank database contains nearly 3000 genomes of Basidiomycota. However, only about 80 genome records belong to species in the Polyporaceae family, with most assembled at the contig level. In recent years, genomes of other Polyporaceae species have been published, such as *Ceriporia lacerata* [54], assembled at the contig level with a genome size of 36 Mb and an N50 of 3.4 Mb. Advances in sequencing technology have also enabled the publication of high-quality chromosome-level genomes for Polyporaceae species, including *Cryptoporus qinlingensis*, which has a genome size of 39.1 Mb and an N50 of approximately 34 Mb for both haplotypes [55]. Nevertheless, chromosome-level genomes remain scarce, and telomere-to-telomere (T2T) assemblies are even rarer. Here, we assembled the *G. frondosa* genome, achieving a size of 35.74 Mb, an N50 of 2.98 Mb, and successful identification of telomeres on all 12 chromosomes. Comparative genomic analysis indicated that the *G. frondosa* CH1 genome assembled in this study shares the highest average nucleotide identity (ANI) of 98.5% with the *G. frondosa* strain GCA003313135.1, and the lowest ANI (96.5%) with strain GCA041464085.1. These ANI values reflect evolutionary divergence among strains, consistent with taxonomic classifications. Although this study presents the first near-complete high-quality genome of *G. frondosa*, high-quality genomic data for the Polyporaceae family remain scarce. In the future, the accumulation of additional high-quality genomes will further enhance species information and associated analyses. In addition, differences in sequencing technologies, assembly methods, and annotation pipelines among the four *G. frondosa* genomes may introduce biases in key metrics such as genome size, gene count, and functional annotations, potentially compromising the reliability of our comparative analyses. These technical variations could obscure subtle genomic differences or exaggerate perceived similarities. This limitation underscores the importance of accounting for technical heterogeneity in comparative genomics. Reducing such discrepancies in future analyses would enhance the accuracy and reproducibility of the results.

*G. frondosa* belongs to the genus *Grifola* within the Polyporaceae family. Previous studies have explored its phylogenetic position using the internal transcribed spacer (ITS) region of nuclear ribosomal DNA (rDNA) and organellar data. For instance, a phylogenetic analysis of 51 commercially valuable *G. frondosa* isolates (21 from eastern North America, 27 from Asia, 1 from Europe, and 2 of unknown origin) revealed distinct species delineation between isolates from eastern North America and Asia [56]. More recently, global phylogenetic analysis of the genus *Grifola* using ITS and β-tubulin (TUBB) sequences identified two major species groups: Northern Hemisphere (NH) and Southern Hemisphere (SH), with *G. frondosa* classified under the NH branch [57]. Additionally, a mitochondrial genome-based study further indicated that *G. frondosa* groups with *Sparassis crispa*, *Laetiporus sulphureus*, *Wolfiporia cocos*, and *Taiwanofungus camphoratus* [58]. However, the absence of whole-genome data has previously precluded a comprehensive genome-wide analysis of its phylogenetic position. In this study, we assembled a high-quality chromosome-level genome of *G. frondosa* CH1 and constructed a phylogenetic tree using 2009 single-copy gene families extracted from the genomes of 11 other fungal species available in public databases. The analysis revealed that the 12 species form three evolutionary clades, with *G. frondosa* CH1 being most closely related to *T. cinnabarina*. Leveraging this robust phylogenetic tree, we estimated that the divergence between *G. frondosa* CH1 and *T. cinnabarina* occurred approximately 105 Mya. Using the time-calibrated tree, we identified and functionally annotated, expanded and contracted gene families in *G. frondosa* CH1, finding significant enrichment in secondary metabolic pathways, such as terpenoid synthesis, which highlights unique biological traits acquired during its evolution. With advances in sequencing technology, whole-genome data have become a mainstream approach for resolving species’ phylogenetic positions. Our findings substantially enhance the reliability of *G. frondosa* CH1’s phylogenetic classification. As more fungal genomes are sequenced in the future, the evolutionary history and relationships of different species will be further refined.

Carbohydrate-active enzymes (CAZymes), essential for carbon acquisition and energy metabolism, were analyzed in the *G. frondosa* genome. A total of 361 CAZyme-related genes were identified, including glycoside hydrolases (GHs, 49%), auxiliary activities (AAs, 23%), glycosyltransferases (GTs, 17%), carbohydrate esterases (CEs, 6%), carbohydrate-binding modules (CBMs, 1%), and polysaccharide lyases (PLs, 4%). Comparative analysis with other *G. frondosa* strains showed that CAZyme family proportions are conserved across strains, though significant expression differences were observed in GTs. GTs are responsible for catalyzing the formation of glycosidic bonds, a crucial step in polysaccharide biosynthesis [59]. These findings suggest that different *G. frondosa* strains vary in their polysaccharide acquisition and production efficiency. Although KEGG enrichment analysis aids in mapping metabolic pathways in fungal genomes, it has notable limitations. The database, biased toward model organisms, inadequately represents fungal-specific pathways, often leading to misannotation of species-specific genes—such as those involved in secondary metabolism—in non-model fungi like *G. frondosa*. Low sequence similarity among fungal genes further complicates homology-based annotation, potentially resulting in inaccurate KO assignments and biased enrichment outcomes. Additionally, KEGG’s static nature lags behind rapidly evolving fungal research, particularly for gene families like P450s and CAZymes. To address this, we validated our results using multiple databases to enhance accuracy, though inherent biases persist. Future studies could leverage fungal-specific databases or multi-omics data to improve KEGG-based analyses.

Transcriptomic analysis under light and dark conditions identified 231 differentially expressed genes (DEGs), of which 131 were upregulated and 88 downregulated. Enriched pathways for these DEGs included “carbon fixation in the Calvin cycle” and “protein processing in the endoplasmic reticulum”. Gene Ontology (GO) enrichment analysis revealed significant terms such as “translation regulation activity”, “catalytic activity”, and “molecular transducer activity”, indicating the activation of light-induced metabolic and biosynthetic processes. A subset of DEGs included CAZymes, particularly glycosyltransferases (GTs, 17%), which suggest that light exposure impacts polysaccharide synthesis in *G. frondosa* CH1. Additionally, some DEGs were linked to the triterpenoid biosynthesis cluster and CYP450 genes, indicating that light exposure enhances the synthesis of triterpenoid metabolites. qRT-PCR validation of 10 randomly selected genes involved in triterpenoid biosynthesis confirmed the transcriptomic results, with minor discrepancies attributed to methodological differences.

This study integrates comparative genomic and transcriptomic approaches to uncover the molecular mechanisms by which light influences anabolic processes in *G. frondosa*. The findings provide valuable insights for the domestication, cultivation, and pharmacological utilization of this economically important species. Additionally, this work advances our understanding of how environmental factors modulate fungal metabolism, with significant implications for biotechnological applications.

## 5. Conclusions

Here, we successfully assembled a nearly complete genome of *G. frondosa* CH1 using Illumina, PacBio HiFi, and Hi-C sequencing technologies. The genome consists of 12 chromosomes, spanning 35.74 Mb, and contains 12,526 protein-coding genes, with high completeness based on the BUSCO assessment. Phylogenetic analysis revealed a close relationship between *G. frondosa* CH1 and *T. cinnabarina*. Transcriptomic analysis showed that light exposure significantly influenced the expression of metabolism-related genes, especially those involved in triterpene biosynthesis. This work provides valuable insights into light-regulated metabolic pathways, offering a foundation for the industrial optimization and bioactive compound production of *G. frondosa*.

## Figures and Tables

**Table 1:** Statistical analysis of the gene structure information of *G. frondosa* CH1.

Characteristics	Value
Total genes	12,526
Avg gene length	1904.79
Avg gene cds length	1297.15
Total cds	74,833
Total cds length	16,248,055
Avg cds length	217.12
Avg cds per gene	5.97
Total exons	78,721
Total exon length	19,626,032
Avg exon length	249.31
Avg exons per gene	6.28
Total genes with introns	11,480
Total introns	66,358
Total intron length	4219,242
Avg intron length	63.58
Avg introns per gene	5.3

## Data Availability

The genome of *G. frondosa* CH1 project has been submitted to GenBank SRA (PRJNA1217597). RNA-seq data have been submitted to GeneBank SRA (PRJNA1217839).

## Supplementary Materials

The following supporting information can be downloaded at: https://www.mdpi.com/article/10.3390/jof11040322/s1, Figure S1: The correlation between cultivation time and colony diameter of *G. frondosa* CH1 under complete darkness and continuous light conditions; Figure S2: qRT-PCR results of the *actin* (*FUN_000155*) gene in *G. frondosa* CH1 under complete darkness and continuous light conditions, where ‘ns’ indicates no significant difference; Figure S3: Hi-C interaction heatmap of chromosome scaffolding in the *G. frondosa* CH1 genome; Figure S4: synteny analysis between the genomes of *G. frondosa* CH1 and GCA041464085.1; Table S1: qRT-PCR primers sequence; Table S2: the Illumina short reads of *G. frondosa* CH1; Table S3: The HiFi reads of *G. frondosa* CH1; Table S4: The Hi-C data of *G. frondosa* CH1; Table S5: The final assembled genome of *G. frondosa* CH1; Table S6: The final chromosome-level assembly genome of *G. frondosa* CH1; Table S7: Classification of repetitive elements in the genome of *G. frondosa* CH1; Table S8: Statistics on small RNAs in *G. frondosa* CH1 genome; Table S9: Statistics on gene function annotation in *G. frondosa* CH1 genome; Table S10: Cluster analysis of 12 fungi gene families based on genomic data; Table S11: Transcriptome sequencing data of mycelium of *G. frondosa* CH1 subjected to 8-day treatment under normal light and dark stress conditions. Table S12: Classification of transporter proteins in the *G. frondosa* CH1 genome; Table S13: The number of each CAZyme category in *G. frondosa* CH1, GCA001683735.1, GCA003313135.1, and GCA041464085.1; Table S14: Classification of secondary metabolic clusters in the *G. frondosa* CH1 genome.
