# Supplementary figures and images for: The Nearly Complete Genome of Grifola frondosa and Light-Induced Genes Screened Based on Transcriptomics Promote the Production of Triterpenoid Compounds
Source: J Fungi (Basel). 2025 Apr 18;11(4):322. doi: 10.3390/jof11040322 (PMC12028448; doi:10.3390/jof11040322)

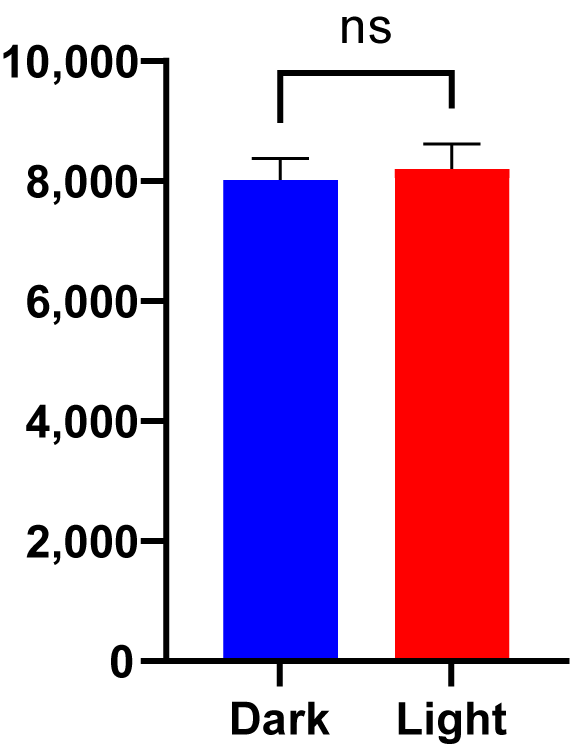

Supplement: Supplementary file 1 [file jof-11-00322-s001.zip › Supplementary File/Figure S2.tif]

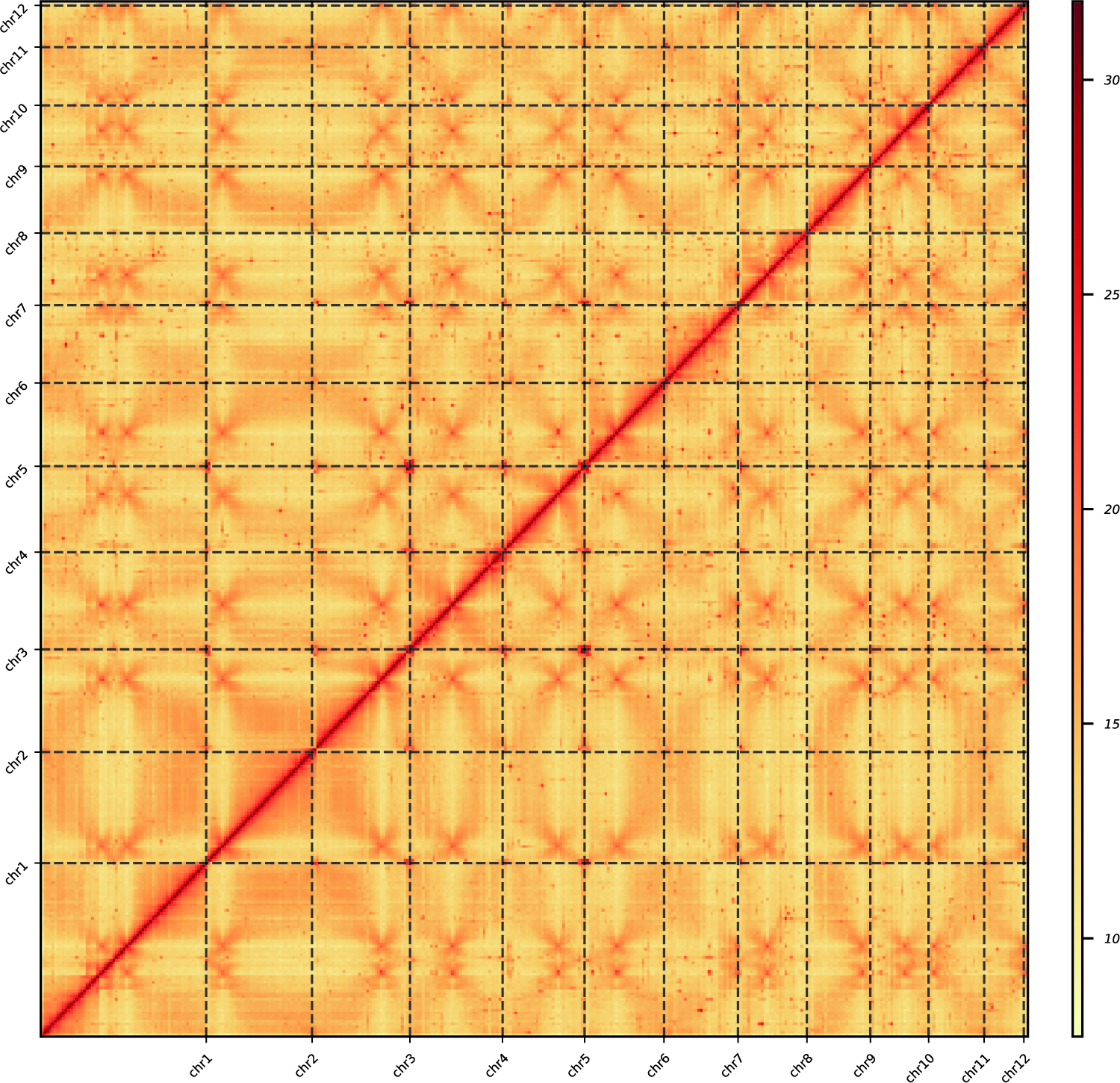

Supplement: Supplementary file 1 [file jof-11-00322-s001.zip › Supplementary File/Figure S3.tif]

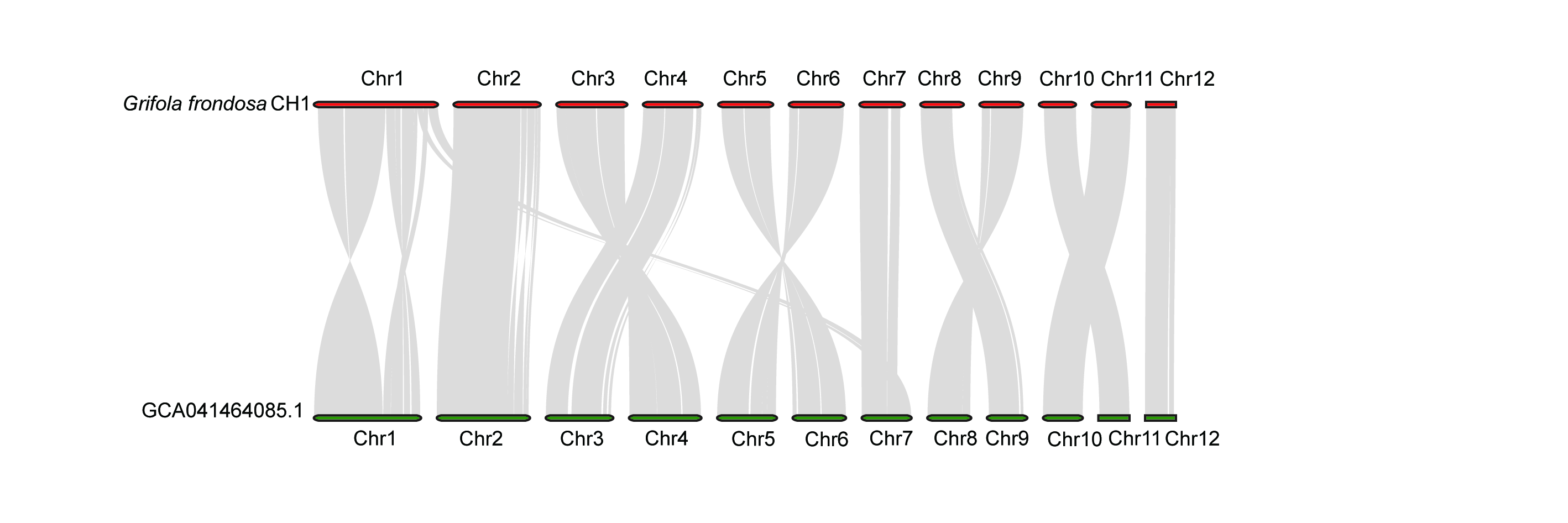

Supplement: Supplementary file 1 [file jof-11-00322-s001.zip › Supplementary File/Figure S4.tif]

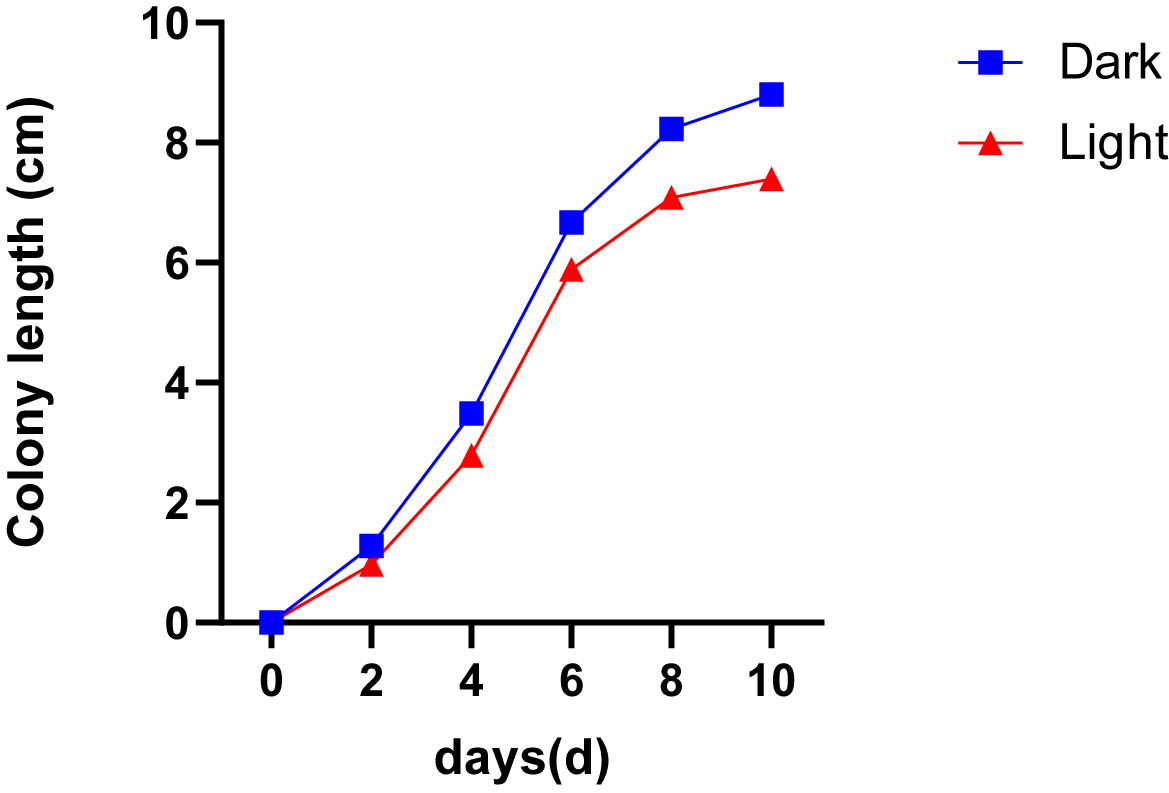

Supplement: Supplementary file 1 [file jof-11-00322-s001.zip › Supplementary File/Figure S1.tif]
